# Correlation between Patent Foramen Ovale, Cerebral “Lesions” and Neuropsychometric Testing in Experienced Sports Divers: Does Diving Damage the Brain?

**DOI:** 10.3389/fpsyg.2016.00696

**Published:** 2016-05-11

**Authors:** Costantino Balestra, Peter Germonpré

**Affiliations:** ^1^DAN Europe Research DivisionBrussels, Belgium; ^2^Faculté des Sciences de la Motricité, Université Libre de BruxellesBrussels, Belgium; ^3^Motor Sciences and Physiotherapy, Environmental and Occupational (Integrative) Physiology, Haute Ecole Paul Henri SpaakBrussels, Belgium; ^4^Center for Hyperbaric Oxygen Therapy, Military Hospital Queen AstridBrussels, Belgium

**Keywords:** adverse effects, SCUBA diving, PFO, embolism, long term effects

## Abstract

SCUBA diving exposes divers to decompression sickness (DCS). There has been considerable debate whether divers with a Patent Foramen Ovale of the heart have a higher risk of DCS because of the possible right-to-left shunt of venous decompression bubbles into the arterial circulation. Symptomatic neurological DCS has been shown to cause permanent damage to brain and spinal cord tissue; it has been suggested that divers with PFO may be at higher risk of developing subclinical brain lesions because of repeated asymptomatic embolization of decompression-induced nitrogen bubbles. These studies however suffer from several methodological flaws, including self-selection bias. We recruited 200 volunteer divers from a recreational diving population who had never suffered from DCS; we then randomly selected 50 of those for further investigation. The selected divers underwent brain Magnetic Resonance Imaging to detect asymptomatic brain lesions, contrast trans-oesophageal echocardiography for PFO, and extensive neuro-psychometric testing. Neuro-psychometry results were compared with a control group of normal subjects and a separate control group for subjects exposed to neurotoxic solvents. Forty two divers underwent all the tests and are included in this report. Grade 2 Patent Foramen Ovale was found in 16 (38%) of the divers; brain Unidentified Bright Objects (UBO's) were found in 5 (11.9%). There was no association between PFO and the presence of UBO's (*P* = 0.693) or their size (*p* = 0.5) in divers. Neuropsychometric testing in divers was significantly worse from controls in two tests, Digit Span Backwards (DSB; *p* < 0.05) and Symbol-Digit-Substitution (SDS; *p* < 0.01). Compared to subjects exposed to neurotoxic solvents, divers scored similar on DSB and SDS tests, but significantly better on the Simple Reaction Time (REA) and Hand-Eye Coordination (EYE) tests. There was no correlation between PFO, number of UBO's and any of the neuro-psychometric tests. We conclude that for uneventful recreational diving, PFO does not appear to influence the presence of UBO's. Diving by itself seems to cause some decrease of short-term memory and higher cognitive function, including visual-motor skills; this resembles some of the effects of nitrogen narcosis and we suggest that this may be a prolonged effect of diving.

## Introduction

SCUBA diving on air exposes divers to possible nitrogen decompression problems upon their ascent and in the hours after the dive. Generally, it is accepted that these problems (DCS: Decompression Sickness) are caused by the formation of gas bubbles in the venous/arterial blood and/or supersaturated body tissues we will refer to circulating bubbles as Vascular Gas Emboli (VGE). Although the precise parameters of bubble formation are not known, and many interfering (facilitating or protective) factors have been described (Carturan et al., 1999; Blatteau et al., 2008; Germonpre et al., 2009), it is also generally accepted that the more “severe” (i.e., saturating) the dive has been, the more risk is present (Eckenhoff and Vann, 1985; Gardette, 1989; Eckenhoff et al., 1990). Decompression “rules” have been developed that, when followed, offer a reasonable protection from DCS. It has been shown however, that even when these rules are followed, nitrogen bubbles can be present in central venous blood, in large enough quantities to possibly cause DCS (Broome, 1996; Marroni et al., 2004; Bennett et al., 2007).

It has been postulated that these bubbles will embolise in the pulmonary vasculature and to a great extent be “filtered out” (after a short period of blockage, the nitrogen and oxygen will diffuse out into the alveolar air). Thus, the lung acts as an efficient “bubble filter,” and only when too much bubbles are present, these could pass into the arterial circulation (Butler and Hills, 1979). In this respect, it is important to limit bubble production (Egi and Gurmen, 2000), and this is possible by using “low-bubble” decompression schedules (Dunford et al., 2002), one of the research areas explored by DAN Europe (Divers Alert Network Europe) during the recent years (Marroni et al., 2004; Bennett et al., 2007).

Arterialisation of gas bubbles (VGE) is possible through other pathways, such as patency of the Foramen Ovale (PFO) of the heart (Moon et al., 1989; Cross et al., 1992; Vik et al., 1993; Bove, 1998, 2014; Gerriets et al., 2000). This condition is present in 25–30% of all humans, as a remainder of the fetal cardiac anatomy (Hagen et al., 1984). It is a “right-to-left” shunt, but it is a popular misconception that a PFO allows the continuous passage of blood between the right and the left atrium.

Firstly, the atrial pressure on the right side of the heart is generally (in 95% of the duration of the cardiac cycle) lower on the right side than on the left side. Since the Foramen Ovale is a valve-like structure, opening from right to left, this pressure differential tends to close the valve, prohibiting a passage of blood and bubbles (Cambier et al., 1993). Secondly, the flow coming from the superior vena cava passes over a tissue fold of the right atrial wall before reaching the PFO (or the Fossa Ovalis). This causes a sudden increase in the rate of the flow, and when it meets the flow coming from the inferior vena cava, a venous turbulence is caused in the right atrium. This turbulence tends to sweep the bubbles away from the interatrial septum. The blood flow coming from the inferior vena cava (where most of the decompression nitrogen bubbles would be present), which is initially directed almost straight to the Fossa Ovalis, is thus diverted away from the PFO (Gin et al., 1993).

These two mechanisms would in natural conditions prohibit venous blood and bubbles to cross the Foramen Ovale. After diving, two other conditions may arise however to facilitate or permit this right-to-left shunt. Continuous pulmonary embolization of nitrogen micro bubbles after a saturating dive will invariably lead to an increase in the pulmonary vascular resistance and a retrograde augmentation of the right atrial pressure. This may suffice to increase the right atrial pressure above the left atrial pressure for sufficient time to allow shunting of blood (Vik et al., 1993). Adding to that, as can be demonstrated during contrast trans-oesophageal echocardiography, certain respiratory maneuvres, by variations in the intrathoracic pressure, cause a temporary reversal of the inter-atrial pressure gradient and thus permit shunting. These maneuvres can be voluntary or involuntary (Cambier et al., 1993; Balestra et al., 1998).

Finally, the timing (after surfacing from a dive) at which right-to-left shunting occurs, plays a role. During the ascent and after surfacing, tissues progressively “desaturate” from their inert gas content, and nitrogen bubbles that embolise in those tissues (e.g., the brain) may well be dissolved without causing any harm. This is due to the so-called “oxygen window,” a phenomenon also responsible for the fact that microbubble contrast echocardiography examinations in normal (non-“supersaturated”) patients do not cause decompression sickness. Some more slowly desaturating areas of the central nervous system may be more “at risk” (Germonpre et al., 2015).

Patency of the Foramen Ovale can be held responsible for a large number of “unexplained” DCS, especially for some types of DCS (cerebral, high-spinal, and cutaneous; Germonpre et al., 1998, 2015; Germonpre, 2005; Gempp and Blatteau, 2009; Blatteau et al., 2011a,b). The “Odds Ratio” for DCS when diving with vs. without a PFO has been calculated from 2.5 (Bove, 1998) to as high as 4.5 for cerebral-type DCS (Germonpre et al., 1998; Germonpre and Balestra, 2004).

Several studies and reports have raised concerns that, even when no clinical signs of DCS are present after a dive, divers with a PFO may arterialise nitrogen bubbles and thus suffer from subclinical brain embolism. This may lead to deterioration in brain performance and detectable brain white matter lesions on MRI (magnetic resonance imaging) (Todnem et al., 1991; Reul et al., 1995; Knauth et al., 1997; Wilmshurst, 1997; Gempp et al., 2008). The exact nature of these “lesions” has not been established as vascular, and they might perhaps best be called “UBO's” (Unidentified Bright Objects). If these findings were confirmed, diving could constitute a serious health hazard for divers with a PFO (Edmonds and Boughton, 1985). There are indications that the presence of white matter lesions leads significantly more frequently to Alzheimer-type dementia and cognitive decline in otherwise healthy people over 60 (Vermeer et al., 2003, 2007).

However, most of the published studies on divers suffer from potentially serious methodological shortcomings, so that their conclusions need verification. After all, diving is a relatively young sport, and although there are already divers who passed seemingly healthy through a 40-year or more diving career, it is only the last 20 years that diving “has come to the masses.” Any long-term health effects may only become noticeable after 10–20 more years (Reuter et al., 1997).

Most of the MRI studies in divers suffer from one of, or all the below selection biases:

1. Divers were asked to participate if they had never suffered from DCS (Knauth et al., 1997), and only their word was taken for that—thus introducing a potential selection bias. Many divers have experienced dizziness or abnormal fatigue after some dives, but never reported this as DCS because of various reasons (Hagberg and Ornhagen, 2003; Brubakk et al., 2014); these may well (subconsciously or not) volunteer for the study to be reassured that all is well.
2. Diving experience and age of the participating divers was extremely varied and dispersed, ranging from 5 to 2500+ dives and from 17 to 55+ years of age—it is easy to see how the normal results from inexperienced divers can “blur” out any abnormal results from long-time divers, even after some stratification for age and dive experience (Todnem et al., 1991; Knauth et al., 1997; Schuchlenz et al., 2002).
3. MR imaging was performed using only T1 and T2 image weighing techniques, possibly categorizing normal variants such as Wirchow-Robin spaces as “abnormal”(Todnem et al., 1991).
4. The finding of abnormal signal spots was not correlated to any psychometric function testing—although it is difficult and time-consuming, it is important to ensure that the observed UBO's correlate with functional abnormalities and are not just morphological curiosities (Reul et al., 1995; Knauth et al., 1997).

## Methods

In order to respond to as many of these concerns as possible, our diver population was carefully selected. The study protocol was approved by the Academic Bioethical Committee for Higher Education of Brussels (B200-2011-117) as compliant with the declaration of Helsinki.

First, divers were asked to volunteer for the study if they corresponded to the following criteria:

1. Diving experience of at least 5 years and at least 200 dives
2. No history of decompression sickness symptoms, treated or not
3. Age maximum 40 years
4. No known cardiovascular or neurological disease

Then, of 200 candidates for the study, 50 were at random selected using a computer-generated randomization list (MS Excel). This way, even if a certain “selection bias” would have been present, at least this bias would be “diluted” 1–4 by the random selection procedure.

All chosen participants signed an informed consent form.

Of these 50, finally 42 passed all the tests. Eight were excluded because they “in fine” refused trans-oesophageal echocardiography (5 out of 8) or no agreement could be reached regarding appointments for the neuro-psychometric testing (3 out of 8).

All divers were subjected to a thorough medical interrogation and physical examination and three tests: contrast trans-esophageal echocardiography (c-TEE), brain MRI imaging and neuro-psychometric testing.

The c-TEE was performed according to a standardized method described previously (Germonpre et al., 1998). In short, after introducing the TEE probe and visualizing the right and left atria, an agitated saline contrast solution (9.5 ml saline mixed in a double-syringe system with 0.5 ml air) was rapidly injected in the right antecubital vein, at the end of a “straining” maneuver performed by the diver. After the arrival of contrast into the right atrium, the number of contrast bubbles passing into the left atrium was observed. Permeability of the Foramen Ovale was assessed using a semi-quantitative method (Grade 0, no bubble passage; Grade 1, less than 20 bubbles; Grade 2, abundant bubble passage during the first three cycles after arrival of contrast in the right atrium; Germonpre et al., 1998; Schuchlenz et al., 2002). During the same examination, a general assessment was made of the cardiac morphology and anatomy, and more specifically of the anatomy of the interatrial septum: incomplete fusion (“double contour”) of the two septal leaflets or aneurysmatic movement of the septum (Germonpre et al., 2005). Contrast-TEE is considered the standard for detection of PFO, if taking into account some technical and methodological details (Attaran et al., 2006; Johansson et al., 2010).

Brain MRI was performed on a separate day using a 1.5 Tesla MRI, and with three sequences (axial T2, sagittal T1, and axial FLAIR) in 5 mm slices. Imaging studies were viewed and analyzed by an independent radiologist, unaware of the results of the PFO testing. An UBO was diagnosed if it appeared at the same time as hyperintense on T2 as well as on FLAIR (Yanagawa et al., 1998; Balestra et al., 2004). Number and size of UBO's were noted, as well as localization.

Neuro-psychometric testing was performed on yet another day, using a computerized testing battery (Neuroscreen, IDEWE, Belgium), a hardware-software implementation of the Neurobehavioral Evaluation System (NES) test (Baker et al., 1985), selecting the most sensitive tests for the early detection of neurotoxicity of solvents exposure in industrial settings (Bulterys, 1993). The NES test battery provides a standardized and precise presentation of test materials and efficient, objective and accurate collection of response data. Cognitive disturbances can be detected at an early stage with neuro-psychometric performance tests (Baker and Letz, 1986; Baker et al., 1988; Echeverria et al., 1991; Anger, 2003).

The Neuroscreen testing program contains four neuro-psychometric performance tests:

- The Simple Reaction Time test (REA): a red square on a joystick lights 60 times with random time intervals (min 2.5 s–max 5.5 s). The test person has to push a button with the index of his writing hand. This test stands for the psychomotor speed (attention and reaction time). The accuracy is up to one hundredth of a second. After processing, five results are stored: the mean reaction time, the standard deviation, the fifth best reaction time, the constant reaction time and the stability of the reaction time. For analysis of subtle neurotoxic symptoms, the stability of the reaction time (REA-Stab) proved to be the most sensitive indicator (Viaene et al., 2001). In this study, both parameters (REA and REA-Stab) were taken as outcome measures.
- The Symbol Digit Substitution test (SDS): here, 9 digits are associated with 9 simple symbols. Underneath that, the 9 symbols are presented in a different order and the test person has to fill in, as quickly as possible, the corresponding digit. This test is performed 5 times with changing combinations. The test is a measure for the attention (perception and coding; visual-motor performance). After processing, four results are stored: the mean time, the fastest time, the mean time for every digit of the best time and the second best time in seconds (SDS1+2mean). From a previous validation study, the SDS1+2mean was selected as the most sensitive indicator (Michiels, 1999; Viaene et al., 2001). The “expected performance” is calculated on the basis of age and scolarity (Education) level, and the procentual difference between expected and achieved performance is taken as the outcome measure.
- The Digit Span Backwards test (DSB): the test person has to enter, in reverse order, the single digits that are presented one by one and at random on the screen. The time interval between each digit is 0.6 s. The test starts with 2 digits and this number is increased every time the answer was correct or decreased when the answer was incorrect. A maximum of 20 digits can be displayed on the screen. This test measures the short-term memory and ability to concentrate. The “expected” performance of the test person is calculated according to age and scolarity level. The difference between expected and observed performance is taken as the outcome measure.
- The Hand-Eye Coordination test (EYE): A sinusoidal curve is presented on the screen. The subject is asked to follow this track with a moving cursor, by means of a joystick. The subject controls only the vertical displacements of the pointer; the (constant) sweeping speed is set by the computer. The test person has to follow, as good as possible, the sine curve presented on the screen. A total of seven runs is done, and for each run, the deviation off the proposed track (missed surface area compared to the sine curve) is calculated in pixels. The best score out of seven runs is stored as the outcome measure.

This particular testing battery was chosen for several reasons:

- It has been developed for measurement of subtle “neurotoxic” effects
- Availability of reference data from a control group of 161 “normal” subjects, and an “exposed” group of 96 persons (industry workers with regular professional contact with potentially neurotoxic solvents; Michiels, 1999)
- Taking into account differences in education and schooling
- Excellent reproducibility of the test results,
- And a fully automated test administration.

The whole testing procedure takes on average 45 min, including a education and “neurotoxicity exposure” questionnaire (the NSC-60; Viaene et al., 2001), and a vocabulary test for determination of general linguistic school level.

Results were analyzed after testing of normality by means of Kolmogorov-Smirnov test; since not all the data were normally distributed, Mann–Whitney *U*-test and Fisher exact test were used, and Spearman correlation coefficients when appropriate, using a statistical package (GraphPad Prism version 6.00 for Windows, GraphPad Software, La Jolla California USA) on the PC. The significance level has been set “a priori” at *p* < 0.05.

## Results

### Demographics

There were 4 female divers and 38 male divers. Mean age was 36 years (SD 4.85 years). Mean Body Mass Index was 25.3. Thirty-four (80%) were non-smokers. Mean years of diving was 11.7 (SD 6.2). Mean number of dives was 620 (SD 465). Of those, 359 (SD 340) or 57.9% were dives shallower than 30 msw, and 91 (SD 93) were dives deeper than 40 msw. Nineteen of the divers had performed nitrox or trimix dives (45%). For those, a mean of 9% of all dives were performed using mixed gases. There was so significant difference between male and female divers for any of these data. Three divers (7%), all male, had a history of arterial hypertension, though only two were on active treatment. As expected, there were no divers who had a history of cardiac or neurological disorders. None of them had at any one point in their diving career suffered from abnormal fatigue, dizziness, visual, or auditive disorders (all of these which may have indicated a decompression sickness episode).

### c-TEE

Patency of the Foramen Ovale was detected in 27 of 42 (64.28%) of the divers. Of those, 16 (38% of the total), were Grade 2 PFO. There were two female divers with PFO (2/4). There was no correlation between the presence of PFO and age (Spearman *r* = 0.18; *p* = 0.76), number of dives performed (Spearman *r* = 0.14; *p* = 0.38), or maneuvres used for middle ear equilibration (Spearman *r* = 0.04; *p* = 0.77). There was however a very weak correlation between dive hours logged and PFO patency (Spearman *r* = 0.32; *p* = 0.03).

### Cerebral MRI

In 5 divers, a total of 5 cerebral UBO's were detected (11.9%). Of those, 3 divers had a PFO (2 a Grade 2, one a Grade 1 PFO). Mean size (area) of the UBO's was 3.8 mm^2^ (SD 1.5 mm^2^). There were three parietally located UBO's and 2 were frontally located. There was no statistical significant difference with regard to presence (*p* = 0.639; Fisher's Exact Test) or size (*p* = 0.5 Mann–Whitney *U*-Test) of UBO's between divers with or without PFO. There was no correlation between grade of PFO and size of UBO (Spearman *r* = −0.017; *p* = 0.918). There was also no correlation between number of dives and presence of UBO's (Spearman *r* = −0.11; *p* = 0.492), or between age (Spearman *r* = 0.13; *p* = 0.7) or smoking habits (pack-years) and presence of UBO's (Spearman *r* = 0.23; *p* = 0.5). Of course, the number of UBO's is very small.

### Neuro-psychometric testing

The results of the Neuroscreen tests were compared between divers with or without PFO (PFO+ and PFO−), and then to the control group of 161 non-diving subjects used in the validation of the test, of which the data were available. Finally, these results were compared to the group of “exposed” persons reported by Michiels (1999). All comparisons were performed with Mann–Whitney *U*-tests for samples comparisons data or Fisher's test when compared to the “exposed group.”

**Figure 1:**
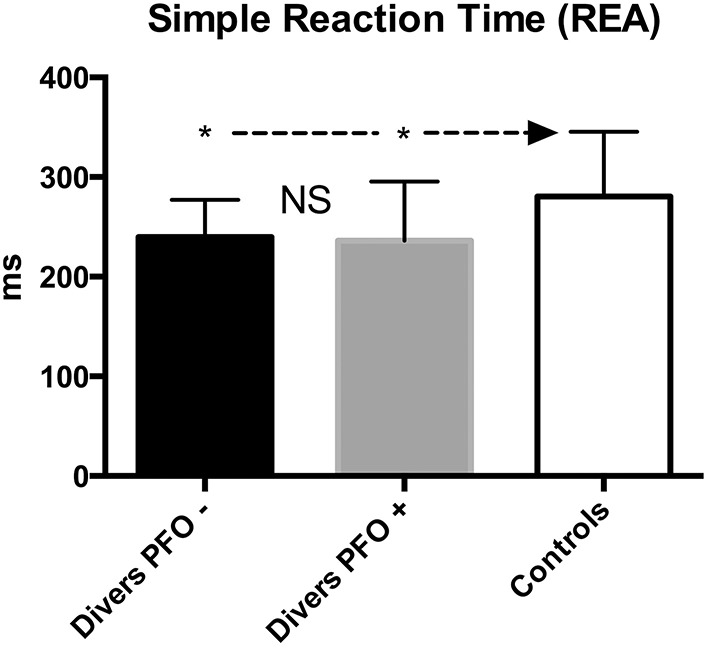
Simple Reaction Time (REA); data are presented as mean and standard deviation bars; ^*^p < 0.05; NS, Not Significant; (Mann–Whitney test).

The Simple Reaction Time test (REA) showed a significant difference between the (better-scoring) divers and the “normal” population (PFO+ vs. PFO−: *p* > 0.5; PFO+ vs. Control: *p* < 0.05; PFO− vs. Control: *p* < 0.05; Figure 1).

The solvent-exposed controls scored significantly worse compared to divers or Controls (*p* < 0.001). The same results were obtained for REA-Stab.

**Figure 2:**
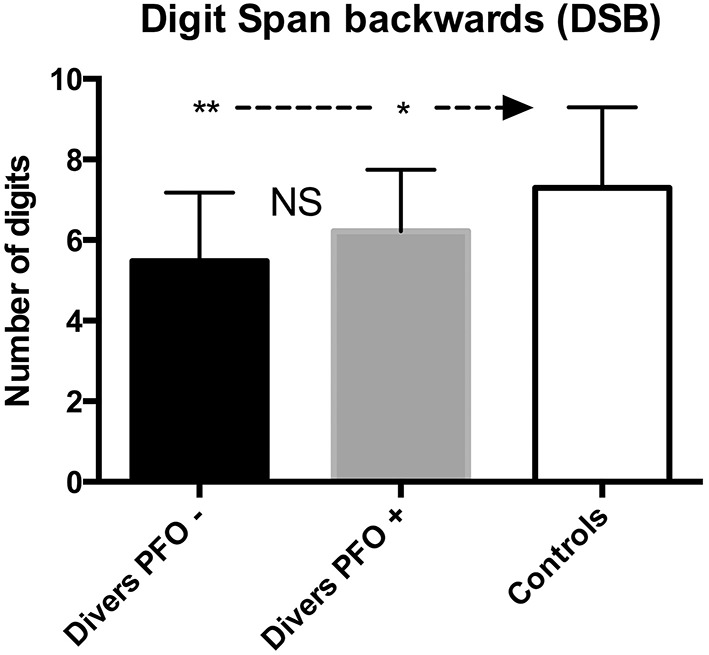
Digit Span Backward (DSB); data are presented as mean and standard deviation bars; ^*^p < 0.05; ^**^p < 0.01; NS, Not Significant; (Mann–Whitney test).

For the Digit-Span Backwards test (DSB), there was a significant difference between the divers (scoring worse) and the Control group (PFO+ vs. PFO−: *p* > 0.05; PFO+ vs. Control *p* < 0.05; PFO− vs. Control: *p* < 0.01; see Figure 2), however, the difference between the divers and the “exposed” group was not significant (*p* = 0.44).

Both groups performed on average 15–25% worse than expected. This test measures the “working memory” and the sustainability of the attention span.

**Figure 3:**
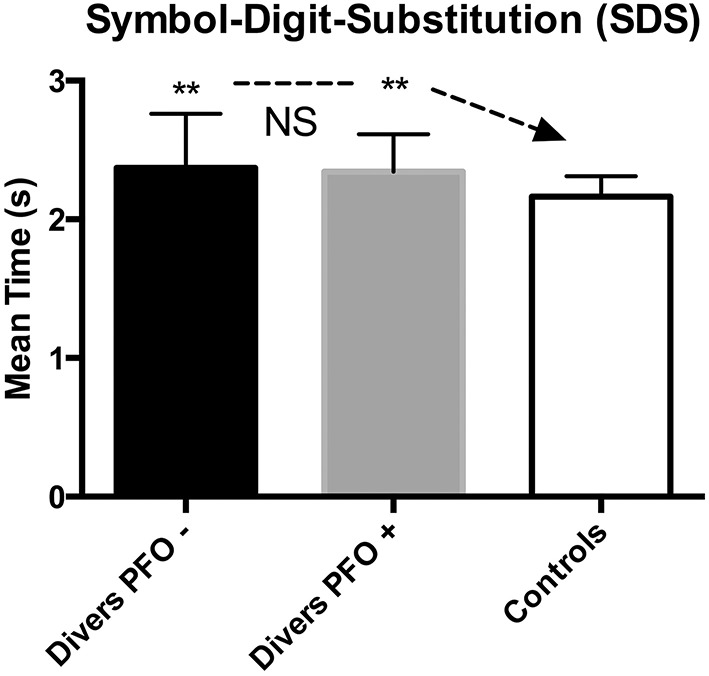
Symbol Digit Substitution (SDS); data are presented as mean and standard deviation bars; ^**^p < 0.01; NS, Not Significant; (Mann–Whitney test).

Likewise, for the Symbol-Digit Substitution (SDS), measuring the visual-motor performance (composed of visual sweeping and visual-spatial attention), a significant difference was found between divers (scoring worse) and Control (*p* < 0.01), whereas there was no difference between divers and the “exposed” group (*p* = 0.29). Here, both groups scored on average 15–30% worse than expected (see Figure 3).

**Figure 4:**
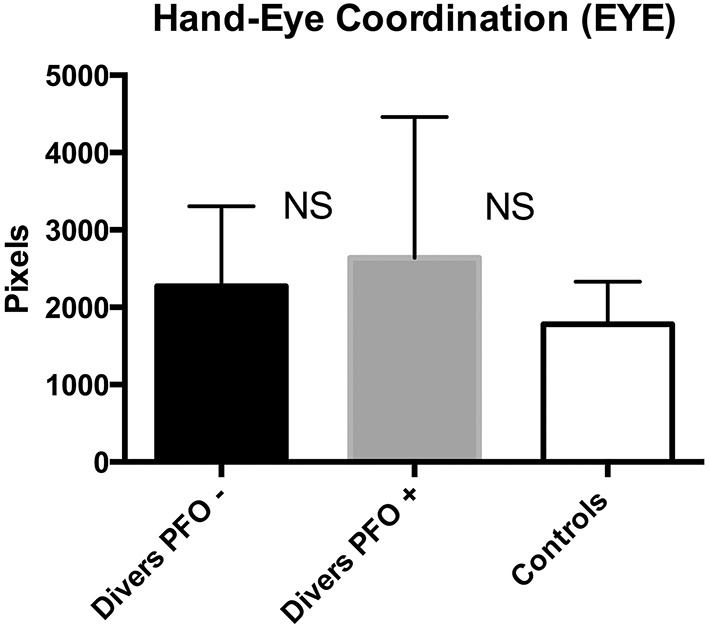
**Hand Eye Coordination (EYE); data are presented as mean and standard deviation bars; NS, Not Significant; (Mann–Whitney test)**.

The Hand-Eye Coordination Test (EYE) did not show a significant difference between both diver groups; divers and the Control group (*p* > 0.5) whereas the solvent-exposed group scored significantly worse (*p* < 0.001; see Figure 4).

There appeared to be no correlation between the diving experience (number of dives, years of diving) and the neuro-psychometric performance (Spearman correlations all *p* > 0.5).

Neuroscreen test result differences between divers with or without UBO's were all non-significant.

## Discussion

The prevalence of PFO in our population is higher than expected from the anatomic and cardiologic literature (Hagen et al., 1984; Fisher et al., 1995). There is no clear explanation for this, although it has been suggested from previous studies, personal observations and one longitudinal follow-up study on PFO, that divers may have a predisposition for “not closing” small PFO's in the course of adult life as other people do (Hagen et al., 1984). This may lead to a larger than average proportion of divers with a large PFO. Moreover, we have proposed recently that closed or only microscopically patent Foramen Ovale's may be “opened-up” by diving or other strenuous intra-thoracic pressure changing activities (Germonpre et al., 2005). This may be responsible for the almost 65% prevalence in healthy divers who had never had DCS.

There was no difference at all with regard to cerebral white matter “lesions” (UBO's) between divers with or without PFO. The prevalence of these UBO's in both groups however is much smaller than what has been found in other similar studies. This may be explained in large part by methodological differences.

Reul et al. (1995) probably included Wirchow–Robin spaces (Jungreis et al., 1988) as “lacunes.” Whereas this does not influence as much his comparative results (both groups have been examined without FLAIR sequence), when taken out of this context, the sur-estimation of cerebral “lesions” may lead to confusion (40 “lesions” discovered in 27 divers). Also, the divers group was composed of non-randomized divers (the first 55 presenting themselves in response to the call for participation). It is well known that (even oligo-symptomatic but repeated) cerebral DCS can lead to MRI abnormalities (Fueredi et al., 1991; Reuter et al., 1997).

Todnem et al. (1991) did not perform FLAIR sequencing, and secondly compared the divers group to a group with a normal MRI examination. Knauth et al. (1997) found a proportion of “lesions” in PFO+ vs. PFO− divers (16% vs. 11%) roughly similar to our findings (8 vs. 5%). A selection bias similar to the study by Reul may have been present, as divers in this study were selected on the basis of a simple “call for volunteers.”

Balestra et al. (2004), using a fractal mathematical analysis of divers' hyperintense brain spots found in MRI imaging failed to prove any correlation between a Patent Foramen Ovale and these spots in asymptomatic divers, although the sample of divers analyzed showed 60% of PFOs. Moreover, the fractal analysis showed that the pattern exhibited by these spots was less suggestive of vascular origin.

Another publication by Cordes et al. (2000) found a large proportion of professional divers with cerebral MRI findings (6 of 24, 25%), but an even larger (however, statistically not significant) proportion of non-divers with “lesions” (42%). They performed neuro-psychometric testing on both groups and found no differences. It appears from these and our results that cerebral “UBO's,” whether diving or non-diving-related in origin, do not necessarily lead to significant neuro-psychometric performance decrements.

It is commonly accepted that white matter spots may be correlated with a reduction of working memory, especially when located in the Frontal area. In our study only two spots were found in this area, the individuals presenting these UBO's were not performing notably worse than the other divers without such “lesions”; it is therefore improbable that this could be invoked to explain differences if present.

The differences that we report here, of the neuro-psychometric test results between divers and non-divers, conform to previous reports by others.

Slosman et al. (2004) reported, in a population of 102 divers, an impairment of the working memory compared to a control group. Tetzlaff et al. (1999) found a significant difference (*p* < 0.01) between divers and a control group on psychometric tests investigating the visual-motor skills. These authors did not find any correlation between these results and the total number of dives nor their depth.

In a study for the British “Health and Safety Executive,” Hickish et al. (Hickish and Hickish, 2002) have administered a neuro-psychometric screening battery test (NES test) to 51 divers having suffered from DCS and compared those to a control group of divers without DCS and with a control group of non-divers. There was a significant difference between DCS-divers and the two other populations, however, the control divers did not differ from the non-diving population. As it is not stated what was the diving experience of the control divers, we cannot state that this study is in accordance or discordance with our results.

The results of our diver population are significantly different from a “normal” control population in three of the four tests: divers scored better with regard to the REA, but worse in DSB and Symbol Digit Substitution tests. Comparing the divers to a group exposed to neurotoxic solvents, the DSB and SDS test results are similar to those obtained by the “exposed” group, however the Reaction Time results and Eye-Hand coordination are significantly better. It is therefore not justified to state that “diving is a neuro-toxic agent”—for that, the divers groups should have had to be completely congruent to the “solvent-exposed” control group (Michiels, 1999). However, the impairment of the working memory (Thompson-Schill et al., 2002) and of the visual-spatial skills is resembling the impairments observed during nitrogen narcosis, which occurs during the dive at depths exceeding 25–30 m (Hemelryck et al., 2013). As it is known that nitrogen, as well as other inert gases, exert an influence on a certain number of membranous neurotransmitter receptors such as dopamine, serotonin and GABA, it is possible to hypothesize that regular diving to “narcotic” depths could diminish, in the long term, the sensitivity of these receptors. This could lead to a (semi-permanent?) deficit in these performances after some time. This finding has been recently confirmed in a direct comparison between recreational divers and non-diving controls (Hemelryck et al., 2014). However, on the basis of our current results, it does not seem possible to establish a correlation between the number of dives, number of deep dives or diving experience in general, and the outcome of DSB and SDS testing.

## Conclusions

Using a randomized sample population of recreational divers who had extensive diving experience without a history or symptoms of decompression sickness, and using carefully selected reliable diagnostic techniques, we were not able to demonstrate a higher prevalence of cerebral MRI abnormalities in divers.

There was a distinct and significant deterioration in the neuro-psychometric performance in the divers group, in the test items measuring working memory and visual-spatial performance. It was not possible to define a relationship between patency of the Foramen Ovale, diving experience and age, and any of these neuro-psychometric findings. The comparison of the results of this neuro-psychometric testing with those of a control group exposed professionally to neuro-toxic solvents, suggests a mechanism similar to nitrogen narcosis in the divers group. This however remains to be verified, and a repeated neuro-psychometric testing would need to be performed to ascertain the permanent nature of these findings.

From these data, we conclude that uneventful recreational diving, with or without PFO, does not seem to have any influence on the prevalence of Unidentified Bright Objects (UBO's), as observed by MRI scanning; the observed effect on the working memory and visual-spatial performance would merit further study and confirmation. As our sample was predominantly male, a potential gender effect could not be evaluated.

## Author contributions

CB and PG conceptualized and designed the research; analyzed and interpreted data; edited and revised manuscript; approved final version of manuscript. They should therefore be considered as co-first authors.

### Conflict of interest statement

The authors declare that the research was conducted in the absence of any commercial or financial relationships that could be construed as a potential conflict of interest.
